# Does the degree of limitation in activities of daily living in geriatric patients influence the amount of care and case management required? An analysis from the RubiN project

**DOI:** 10.1186/s12877-025-05855-3

**Published:** 2025-04-07

**Authors:** Simone Gloystein, Heiko Krause, Sonja Laag, Neeltje van den Berg

**Affiliations:** 1 Institute for Community Medicine, Section Epidemiology of Health Care and Community Health, University Medicine Greifswald, Greifswald, Germany; 2 Department for Product Strategy/ Development, BARMER Health Insurance, Wuppertal, Germany

**Keywords:** Geriatric patients, Age, Physician networks, Geriatric assessment, Activities of daily living, Intensity of care and case management

## Abstract

**Background:**

The number of people in Germany over the age of 70 will increase significantly over the next 10 years. This will be accompanied by an increase in geriatric diseases and disabilities. An important goal for many geriatric patients is to remain in their own homes for as long as possible, while making use of support services. To achieve this, patients with limitations in their daily activities and in need of geriatric care should be identified as early as possible. The RubiN project implemented assessment-based care and case management for geriatric patients in physician networks in Germany. To support future planning, the present analysis investigated whether the intensity of case management increases with increasing limitations in patients’ activities of daily living.

**Methods:**

Using the Barthel Index, an assessment tool to record patients’ ability to perform activities of daily living, patients’ current limitations were assessed for ten activities (eating; sitting up and moving; washing; toileting; bathing/showering; getting up and walking; climbing stairs; dressing/undressing; bowel incontinence; urinary incontinence). For each item, the score (0 to max. 15 points) is determined and added to the Barthel Index score (max. 100 points). Counselling and coordination services provided by case managers were documented according to medical, nursing, therapeutic and social counselling content. Linear multivariate analysis was performed to determine whether the Barthel Index score was a determinant of the intensity of care and case management.

**Results:**

Two thousand three hundred six patients in the RubiN intervention group (65% female; mean age 81.5 years (SD 5.6)) were included in the analysis. 74% of these patients achieved a Barthel Index score between 100 and 85 points at baseline, and correspondingly, 26% of the patients had a Barthel Index score of 80 points or less. Problems with ‘bathing/showering’, ‘getting up and walking’, ‘climbing stairs’, ‘dressing/undressing’ and ‘controlling urination’ were the most common reasons for not achieving the maximum Barthel Index score of 100 points. A total of 26,833 patient contacts were documented by the care and case manager. On average, patients received 11.6 contacts (SD = 9.1). Social (31.8%) and medical (26.3%) counselling and coordination services were provided in the majority of contacts. “Therapeutic counselling content” and “nursing counselling content” were provided in 21.7% and 20.1% of contacts, respectively. Multivariate analysis showed a significant correlation between an decreasing Barthel Index and a higher number of contacts with the care and case manager.

**Conclusions:**

The Barthel Index score can be used to predict the intensity of care and assistance needed by geriatric patients. The scores provide a good basis for planning and implementing care and case management.

**Trial registration:**

German Clinical Trials Register, ID: DRKS00016642. Registered on 29.10.2019—retrospectively registered.

## Background

The age structure of the German population is changing as a result of the stagnating birth rate and the simultaneous increase in life expectancy. The number of people aged over 70 will continue to rise over the next ten to twenty years, as Germany’s currently most populous cohorts (birth cohorts from 1959 to 1968 (baby boomers) [1]) will then have reached the group of over 70 year olds. Today’s share of the population of over 70 year olds (2022: 16% (13.7 million)) will already have increased by 5.7% in the next ten years (2032: 19% (16.0 million)) and will reach a peak of 18.6 million people (23%) after another ten years in 2042 [2].

In addition to the significant increase in the number of people of retirement age, demographic change is also leading to a growing demand for health and care services. The sharp increase in the proportion of ‘very old’ people is also accompanied by a change in the prevalence of typical diseases in this rapidly growing group of over 70-year-olds. There is an increase in the number of patients, especially with regard to age-related chronic diseases and multimorbidity [3, 4]. These include diseases with a pronounced age association, such as pneumonia, macular degeneration, dementia, joint disease and hypertension. For example, the prevalence of diabetes mellitus in the German population is expected to increase by 20% and the prevalence of visual impairment by 41% by 2050 compared to 2007 [5]. Elderly patients suffering from multiple and often chronic diseases are often also affected by functional limitations that make it difficult or impossible to remain in their own homes. The challenge for the healthcare system is therefore to provide integrated and patient-centred care. This can be achieved through interdisciplinary cooperation between general practitioners, specialists, nurses and therapists, the use of technological innovations such as telemedicine and digital patient records, the adaptation of care and rehabilitation services to the needs of older, multimorbid patients and, above all, better coordination of care (e.g., care and case management (CCM)).

As medical care for the elderly is primarily provided by general practitioners, they should also play a central role in the early identification of functional limitations (e.g. mobility, cognition, self-care). Early identification allows for targeted interventions and improves care in the context of CCM. CCM combines two approaches: care management, which involves the structural and organisational management of care for older patients, and case management, which focuses on the individual, patient-centred coordination of care. Both approaches are complementary. Current general practitioners practice includes routine anamnesis and physical examination of patients, the use of screening tools for early detection, listening to family members’ descriptions of daily behaviour, and early referral to other professionals such as specialists, physiotherapists, occupational therapists or geriatricians. In 2005, geriatric assessments were introduced as a reimbursable service of general practitioners in the German health care system [6]. These are standardised procedures for the comprehensive assessment of the health status of older people. The aim is to identify physical, mental, functional and social limitations at an early stage and to develop individualised treatment and care plans to promote and maintain the quality of life and independence of older patients. However, there are often seemingly insurmountable challenges. These include (1) a lack of time and human resources for complex assessments and coordinated care of older patients by general practitioners, (2) a lack of standardisation of screening tools, (3) patient-related barriers, such as fear of stigmatisation after admitting limitations, (4) fragmented care structures, such as a lack of networking between stakeholders or a lack of clear understanding of patients’ needs. In order to facilitate general practitioners’ ability to identify early indications of functional limitations and thereby contribute to the provision of coordinated, holistic care for older patients, the utilisation of a well-structured CCM could prove beneficial.

CCM is intended to pave the way and create structures for appropriate healthcare for geriatric patients with their often very complex and individual needs for help on a case-by-case (case) and systematic basis (care) by organising and thus coordinating the individual needs of the patients. The aim is to improve or stabilise the patients’ living situation in their own homes, in addition to a possible improvement in health care [7]. In the past, the use of so-called patient guides or case management for vulnerable patient groups with very complex care needs has been investigated and already implemented in several projects. The range of concepts that have been or are being implemented in model projects extends from the use of patient guides for acute diseases such as stroke or myocardial infarction to the use of guides or case managers for chronic diseases such as diabetes or rheumatism [7]. The patient guide is a person or resource who helps patients to orientate themselves in the healthcare system and coordinate the individual care process. The term ‘guide’ clarifies the function: patients are guided safely and purposefully through complex care structures.

The positive effects of such a central support person or resource, both for patients themselves and for their relatives or carers, have been demonstrated several times. For example, the evaluation of the German AGnES projects showed a high acceptance of the delegation of certain medical tasks to qualified personnel among general practitioners, patients and the AGnES staff themselves [8]. In [9], evidence was found that CCM can also help to identify, alleviate or prevent family caregiver burden. The concept of CCM for geriatric patients, implemented in physician or care networks, has already been shown to improve the use of necessary care services for people with dementia [10] and to be a beneficial model in terms of quality of life [11].

A geriatric patient is characterised not only by advanced age (70 years and older), but also by multimorbidity (the simultaneous presence of several acute and chronic diseases), which may be somatic, cognitive or affective. The so-called ‘Geriatric I’s’ are common. This is a classic concept in geriatrics that describes typical problems and syndromes beginning with the letter ‘I’. Recording the ‘I’s’ can provide guidance for prevention, holistic diagnosis and treatment of geriatric patients. The main ‘Geriatric I’s’ are Immobility, Irritability, Instability, Incontinence, Isolation, Intellectual Decline, Insomnia, Impotence, Inappetence, Immunodeficiency, Instable Polypharmacy and Iatrogenic Damage, which often occur in combination and have a significant impact on the daily lives of older people. In addition, a large proportion of geriatric patients suffer from cerebrovascular-neurological (stroke, Parkinson’s disease), cardiovascular (heart failure, peripheral arterial disease), musculoskeletal (fractures after falls, osteoporosis) and other internal diseases. Incontinence, visual impairment, hearing loss, cognitive impairment, depression, pain, dizziness, and increased or decreased body mass index [12] are often associated with multimorbidity, leaving geriatric patients at risk of losing their independence, becoming dependent or requiring long-term care, and, most importantly, experiencing a deterioration in their quality of life. It is necessary to consider the overall condition of patients with the aim of maintaining autonomy in daily life and the best possible health for as long as possible [12].

This paper examines whether and how limitations in activities of daily living in geriatric patients influence the intensity of CCM. The aim of this analysis is therefore to show that it is possible to predict the need for CCM in geriatric patients on the basis of their limitations in activities of daily living, and that this can lead to improved resource planning on the part of CCM.

## Methods

To answer the research question, data from the project “RubiN—continuous care in regional networks” [13] were used.

### Study design

RubiN is a prospective, controlled intervention study designed to investigate whether an assessment-based CCM, implemented in physician networks, leads to an improvement in the care situation and health status of geriatric patients still living at home. In the RubiN study, patients were recruited non-randomly from eight German physician networks certified under Sect. 87b of the German Social Security Code V (SGB V). Patients from five physician networks were allocated in the intervention group. This group implemented the CCM adapted to the specific regional conditions, and the care and case managers were specially qualified according to the GeriNurse curriculum, which was developed from the GeriNeTrainer curriculum specifically for cross-sectoral CCM in geriatrics [14] (Kasprick L, et al: Musterfachcurriculum Geriatrie – GeriNurse – sektorenübergreifendes Care- und Casemanagement 2018, unpublished). The patients from three other physician networks formed the control group and received care for geriatric patients according to the regular standards (“care as usual”). Detailed information on the RubiN project can be found in the design paper for this study [13].

In order to identify health problems, limitations and remaining resources of geriatric patients, patients over 70 years of age were screened in the general practices of the participating physician networks using the Angelina screening tool [14]. Angelina is a self-report questionnaire that provides a low-threshold first impression of patients’ support needs by querying seven geriatric dimensions: housing/need for assistance; medication; mobility; senses; hospitalisation; cognition and mood. A score of 0 to a maximum of 9 points can be achieved. The higher the score, the higher the need for geriatric care. With an Angelina score of ≥ 2 (calculated from at least two of the seven dimensions), patients were included in the study and received an initial geriatric assessment (baseline). This very comprehensive assessment was used to measure outcomes both at patient level and at the level of patient relatives. For example, the following instruments were used to assess mobility (Timed Up and Go, TUG [15]), cognition (DemTect [16]), nutritional status (Minimal Nutritional Assessment, MNA [17]) and quality of life (WHOQOL-OLD [18], WHOQOL-BREF [19]).

The primary patient-level endpoint of the RubiN study was the ability to perform activities of daily living, operationalised by the Barthel Index (BI) [20]. The BI is a questionnaire that systematically records activities that are part of the patient’s daily needs, such as eating, dressing, personal hygiene and toileting.

Due to its ease of use and quick implementation, the BI is one of the most widely used tools for measuring self-care and self-care skills in daily life and is a standard tool in the care context, particularly in geriatrics [21]. In addition to its ease of use, the BI is also characterised above all by its good reliability (ensured by standardised application and high interrater consistency) and validity (achieved by the well-founded mapping of functional everyday activities and the ability to predict needs).

The BI used in this project corresponds to the short version of the Hamburg Manual [21]. The geriatric patients in the RubiN project were assessed with the BI at three time points (baseline, 12-month follow-up and 21-month follow-up). The BI scores obtained at baseline (first time point) are relevant for the present paper.

The extent of the geriatric patients’ self-care ability with regard to the “Activities of Daily Living” (ADL) was assessed with the following 10 items: eating, sitting up and moving, washing, toileting, bathing/showering, getting up and walking, climbing stairs, dressing/undressing, bowel continence and urinary continence.

There are various approaches to analysing the results of the BI and the following methods were used in this study:

- Calculation of the sum score: For each item query, the number of points obtained (0–15) is determined and added together to form a score. A maximum of 100 points (completely independent) and a minimum of 0 points (completely dependent) can be achieved. A higher score indicates greater independence in activities of daily living.
- Categorisation into ‘restriction’ vs. ‘no restriction’: In addition to the overall BI score, the BI was disaggregated according to the presence of restrictions for each BI item (yes = maximum score not achieved or no = maximum score achieved).
- Categorisation in dependency levels: The BI was also analysed according to the following BI score categories (dependency levels) [21]:
  0-30 points (largely dependent on care)
  35-80 points (in need of help)
  85-95 points (selectively in need of help)
  100 points (completely independent)

Once enrolled in the RubiN project, patients underwent a comprehensive geriatric assessment in their homes, including assessments of mobility (Timed Up and Go, TUG), cognition (DemTect), nutrition (Minimal Nutritional Assessment, MNA), management of activities of daily living (IADL) and quality of life (WHOQOL-OLD, WHOQOL-BREF).

### Intervention

Based on the results of this assessment, an individual support plan was developed for the patients in the intervention networks. This means that the necessary and appropriate services from the available regional services (professional and voluntary) were selected and coordinated by a care and case manager in consultation with the general practitioner and the patient and relatives, based on the identified need for support and the existing family and social situation. The individual care plan required the care and case managers to work across professions, sectors and social codes [22]. Each contact of the care and case managers with the patients was documented. In addition to the duration, location and reason for the consultation, the content of the consultation was categorised as medical (e.g. medication check, blood pressure or blood sugar measurement), nursing (e.g. initiation of an application for a degree of care or home nursing care), therapeutic (e.g. application for physiotherapy or occupational therapy) and social (e.g. provision of information on social activation or self-help groups). To ensure standardised and meticulous documentation of contacts between patients and care and case managers, the care and case managers were also trained in this as part of their ‘GeriNurse’ training, which was completed in preparation for the RubiN study (Kasprick L, et al: Musterfachcurriculum Geriatrie – GeriNurse – sektorenübergreifendes Care- und Casemanagement 2018, unpublished).

### Data collection and analysis

The description of patient data (sociodemographic data, BI score at baseline and patient-related documentation for CCM between baseline and 12-month follow-up) was carried out using means with standard deviations for continuous and normally distributed variables and absolute frequencies and percentages for categorical variables. The patient data and analyses presented herein were derived exclusively from those patients for whom all the variables involved had valid values. This was ensured by applying the ‘listwise case exclusion’ method to address any missing values. A multiple linear regression model was used to investigate whether there was a correlation between the outcome variable, the number of coordination and support services provided (= number of CCM documents) per patient and their initial BI score. In addition to the BI score, other predictors were included in the model as independent variables. Consideration was given to which independent variables correlated strongly with the target variable and which independent variables did not correlate strongly with each other (multicollinearity). After applying a stepwise selection procedure (combination of forward and backward selection), an optimal combination of predictors was found. In addition to the BI score, 8 further potential predictors were included in the multiple linear regression model (age, gender, education (school, occupation), living situation (place of residence, housing situation), social support and financial situation). JMP Pro 17 statistical software (SAS Institute Inc.) was used for descriptive statistics and SAS Enterprise Guide® 8.3.7 software (SAS Institute Inc., Cary, NC, USA) for analytical statistics.

## Results

A total of 4,489 patients were recruited for the RubiN study in the general practices of the respective networks. Of these, 3,418 patients were assigned to the 5 intervention networks (intervention group) and 1,071 patients were assigned to the 3 control networks (control group).

**Fig. 1:**
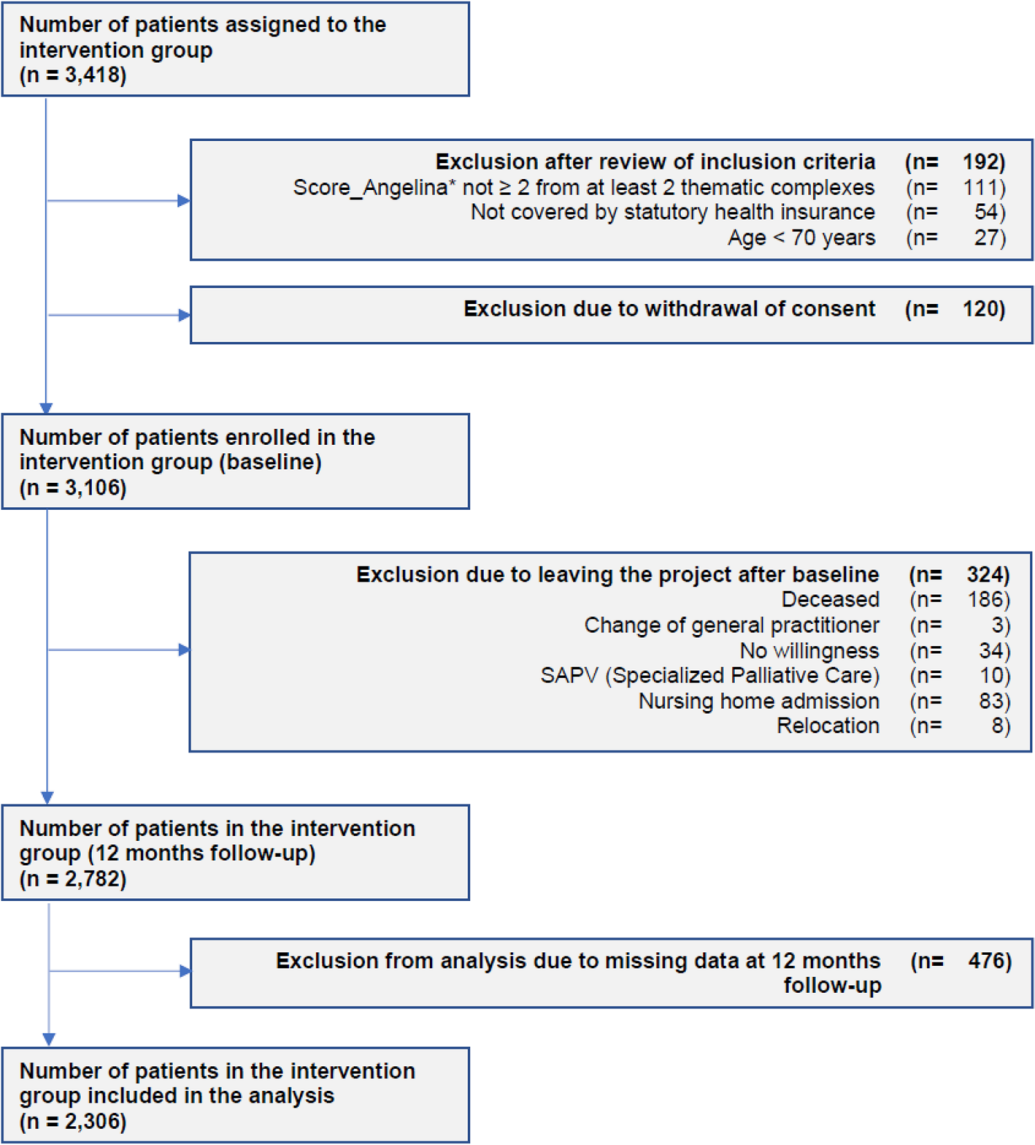
Development of the number of patients in the intervention group from allocation to the analysis performed in this paper

Only the intervention group is of interest for answering the question investigated in this paper, namely whether it is possible to predict the need for CCM in geriatric patients on the basis of their limitations in activities of daily living and whether this can lead to improved resource planning on the part of the CCM. Figure 1 shows the flowchart for the analysis.

Of the 3,418 geriatric patients assigned to the intervention group, 2,306 could be included in the analysis.

### Descriptive statistics

The results of the descriptive analysis for the patients in the intervention group with regard to their socio-demographic characteristics (gender, age, living situation, education, financial situation) and social support are presented in Table 1. The mean age of the patients was 81.5 years (SD = 5,6) and 65% were female. 64% of patients were in the older age group (≥ 80 years). This distribution was similar for both male and female patients. 73% of patients had a lower level of education. 43% of patients lived in rural areas, 41% lived alone at home, 11% reported having no social support and 80% described their financial situation as adequate.

**Table 1:** Sociodemographic characteristics at baseline (*n* = 2,306)

Variable	Total		Male			Female			*p*-Value
	*n*	(%)	*n*	(%)		*n*	(%)		
Number of patients	2,306	(100)	815	(35.34)	(100)	1,491	(64.66)	(100)	
Age; mean age in years (standard deviation)	81.50 (5.64)		81.54 (5.57)			81.48 (5.68)			0.7850^1^
Age groups									0.9621^2^
70–79	839	(36.38)	296	(36.32)		543	(36.42)		
≥ 80	1,467	(63.62)	519	(63.68)		948	(63.58)		
Level of Education									
a.) Educational level									0.1473^2^
low	1,683	(72.98)	580	(71.17)		1,103	(73.98)		
high	623	(27.02)	235	(28.83)		388	(26.02)		
b.) Professional qualification									< .0001*^2^
low	1,758	(76.24)	564	(69.20)		1,194	(80.08)		
high	548	(23.76)	251	(30.80)		297	(19.92)		
Housing situation									
a.) Place of residence									0.1897^2^
rural	999	(43.32)	368	(45.15)		631	(42.32)		
urban	1,307	(56.68)	447	(54.85)		860	(57.68)		
b.) Home situation									< .0001*^2^
living alone	956	(41.46)	172	(21.10)		784	(52.58)		
not living alone	1,350	(58.54)	643	(78.90)		707	(47.42)		
Social support									0.9537^2^
no social support	245	(10.62)	87	(10.68)		158	(10.60)		
social support	2,061	(89.38)	728	(89.32)		1,333	(89.40)		
Financial situation									0.0054*^2^
Finances not adequate	462	(20.04)	138	(16.93)		324	(21.73)		
Finances adequate	1,844	(79.96)	677	(83.07)		1,167	(78.27)		

Data: means and standard deviations for continuous variables, *M* Mean, *SD* Standard deviation; percentages for categorial variables, rounded down/up if applicable – missing values were excluded prior to the calculation of each variable; *p* values for differences between male and female group

^1^two-sided pooled t-test, ^*^p < *0.05*

^2^Chi^2^-tests, *p <* 0.05*

### BI value description

Table 2 shows the distribution of the BI-scores, the presence of BI-items-restrictions (yes or no) and the BI-score-categories achieved at baseline for patients in the intervention group. The most common score was 100 (*n* = 933; 40.46%) and the fewest patients had a BI score of 0 or 5 points (poor geriatric health status) (both *n* = 2; 0.09%). All patients who scored below a BI score of 100 are restricted in at least one BI item. Table 2 also shows in which items in particular there were limitations. Approximately one-third of patients (30 to 36%) have limitations in one of the four areas (BI items) “bathing/showering”, “getting up and walking”, “climbing stairs” and “urinary incontinence”. 22% of patients are restricted in “dressing and undressing” and around 10% (8.7 to 13%) have problems with “eating”, “sitting up and moving”, “washing themselves”, “using the toilet” and “bowel continence”. Regarding the BI score categories (see also Table 2), it can be seen that 40.46% of the patients are assigned to the category “completely independent”, 33.92% to the category “selectively in need of help”, 23.99% to the category “in need of help” and 1.65% to the category “largely dependent on care”.

**Table 2:** BI scores, BI item restrictions and BI score categories at baseline (*n* = 2,306)

Variable	Total		Male		Female		*p*-Value
	*n*	(%)	*n*	(%)	*n*	(%)	
Number of patients	2,306	(100)	815	(35.34) (100)	1,491	(64.66) (100)	
BI score							0.0195*^2^
0	2	(0.09)	1	(0.12)	1	(0.07)	
5	2	(0.09)	2	(0.24)	0	(0.00)	
10	3	(0.13)	2	(0.24)	1	(0.07)	
15	6	(0.26)	2	(0.24)	4	(0.27)	
20	3	(0.13)	1	(0.12)	2	(0.13)	
25	10	(0.43)	6	(0.74)	4	(0.27)	
30	12	(0.52)	7	(0.86)	5	(0.34)	
35	15	(0.65)	6	(0.74)	9	(0.60)	
40	17	(0.74)	5	(0.61)	12	(0.81)	
45	23	(1.00)	7	(0.86)	16	(1.07)	
50	28	(1.21)	14	(1.72)	14	(0.94)	
55	32	(1.39)	15	(1.84)	17	(1.14)	
60	55	(2.38)	26	(3.19)	29	(1.94)	
65	64	(2.78)	26	(3.19)	38	(2.55)	
70	73	(3.17)	26	(3.19)	47	(3.15)	
75	103	(4.47)	30	(3.68)	73	(4.90)	
80	143	(6.20)	39	(4.78)	104	(6.98)	
85	177	(7.68)	57	(6.99)	120	(8.05)	
90	238	(10.32)	75	(9.20)	163	(10.93)	
95	367	(15.92)	112	(13.74)	255	(17.10)	
100	933	(40.46)	356	(43.68)	577	(38.70)	
BI item restrictions (yes or no)							
1. eating							< .0001*^1^
yes	270	(11.71)	128	(15.71)	142	(9.52)	
no	2,036	(88.29)	687	(84.29)	1,349	(90.48)	
2. sitting up and moving							0.0486*^1^
yes	245	(10.62)	101	(12.39)	144	(9.66)	
no	2,061	(89.38)	714	(87.61)	1,347	(90.34)	
3. washing themselves							0.0004*^1^
yes	306	(13.27)	137	(16.81)	169	(11.34)	
no	2,000	(86.73)	678	(83.19)	1,322	(88.66)	
4. using the toilet							< .0001*^1^
yes	200	(8.67)	98	(12.02)	102	(6.84)	
no	2,106	(91.33)	717	(87.98)	1,389	(93.16)	
5. bathing/ showering							0.1352^1^
yes	727	(31.53)	273	(33.50)	454	(30.45)	
no	1,579	(68.47)	542	(66.50)	1,037	(69.55)	
6. getting up and walking							0.2774^1^
yes	743	(32.22)	251	(30.80)	492	(33.00)	
no	1,563	(67.78)	564	(69.20)	999	(67.00)	
7. climbing stairs							0.2120^1^
yes	831	(36.04)	280	(34.36)	551	(36.96)	
no	1,475	(63.96)	535	(65.64)	940	(63.04)	
8. dressing and undressing							< .0001*^1^
yes	513	(22.25)	225	(27.61)	288	(19.32)	
no	1,793	(77.75)	590	(72.39)	1,203	(80.68)	
9. bowel continence							0.6902^1^
yes	202	(8.76)	74	(9.08)	128	(8.58)	
no	2,104	(91.24)	741	(90.92)	1,363	(91.42)	
10. urinary continence							< .0001*^1^
yes	703	(30.49)	201	(24.66)	502	(33.67)	
no	1,603	(69.51)	614	(75.34)	989	(66.33)	
BI score cathegories							0.0015*^2^
1. (0–30) largely dependent on care	*3*8	(1.65)	21	(2.58)	17	(1.14)	
2. (35–80) in need of help	553	(23.98)	194	(23.80)	359	(24.08)	
3. (85–95) selectively in need of help	782	(33.91)	244	(29.94)	538	(36.08)	
4. (100) completely independent	933	(40.46)	356	(43.68)	577	(38.70)	

Data: means and standard deviations for continuous variables, *M* Mean, *SD* Standard deviation; percentages for categorial variables, rounded down/up if applicable – missing values were excluded prior to the calculation of each variable; *p* values for differences between male and female group

^1^two-sided pooled t-test,^*^p < 0.05

^2^Chi^2^-tests, ^*^p < 0.05

### CCM contacts description

The intervention, which included CCM for the 2,306 patients in the intervention group between baseline and the 12-month follow-up, was documented with a total of 26,833 counselling and coordination contacts (see Table 3). This corresponds to an average of 11.64 contacts per patient (SD = 9.08), with a minimum of 0 and a maximum of 97 contacts per patient. A total of 46,013 counselling and coordination topics (multiple answers were possible) were documented. Case managers most frequently provided counselling and coordination services in the social domain (*n* = 14,640; 31.82%), followed by medical topics (*n* = 12,118; 26.34%). In the therapeutic area, *n* = 9,996 (21.72%) and in the nursing area, *n* = 9,259 (20.12%) case manager services were documented.

**Table 3:** Data on patient-related counselling and coordination documentation by CCM (*n* = 2,306)

Variable	Total		Male			Female			*p*-Value
	*n*	(%)	*n*	(%)		*n*	(%)		
Number of patients	2,306	(100)	815	(35.34)	(100)	1,491	(64.66)	(100)	
Number of contacts	26,833	(100)	8,907	(33.19)		17,926	(66.81)		0.0052*^1^
Average number of contacts (standard deviation)	11.67 (9.07)		10.96 (8.70)			12.05 (9.25)			
Counselling content	46,013	(100)	15,913	(34.58)	(100)	30,100	(65.42)	(100)	
medical	12,118	**(26.34)**	4,268	**(26.82)**		7,850	**(26.08)**		0.9299^1^
therapeutic	9,996	(21.72)	3,405	(21.40)		6,591	(21.90)		0.3635^1^
nursing	9,259	(20.12)	3,124	(19.63)		6,135	(20.38)		0.2551^1^
social	14,640	**(31.82)**	5,116	**(32.15)**		9,524	**(31.64)**		0.7296^1^

Data: means and standard deviations for continuous variables, *M* mean, *SD* standard deviation; percentages for categorial variables, rounded down/up if applicable – missing values were excluded prior to the calculation of each variable; *p* values for differences between male and female group

^1^two-sided pooled t-test, *p < 0.05

^2^Chi^2^-tests, ^*^p < 0.05

**Fig. 2:**
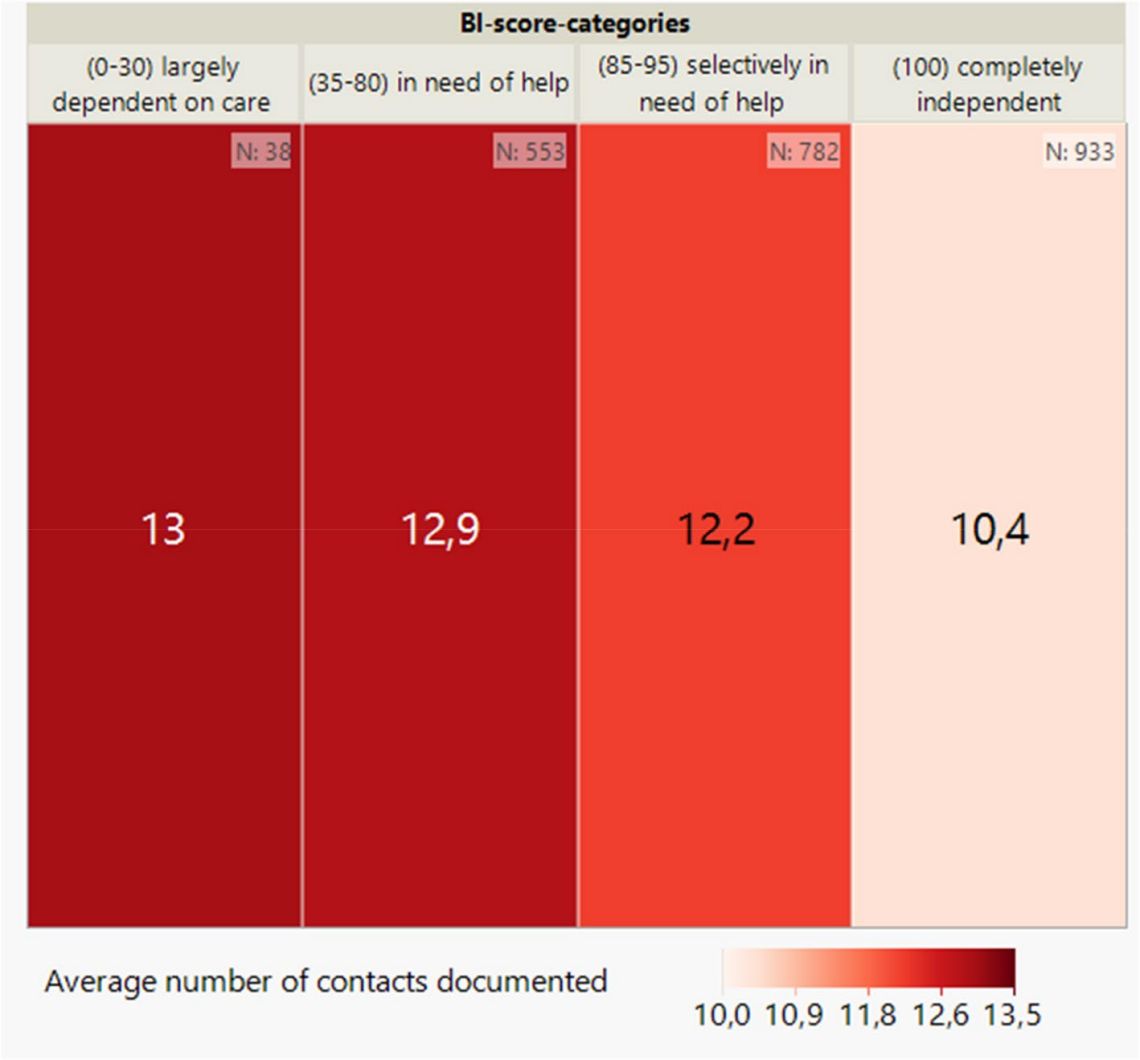
Average number of documented contacts per patient by BI score (categorised) at baseline (*n* = 2,306) shown on a heatmap

The analysis of the distribution of the average number of contacts between case managers and patients depending on the BI score category shows that there is an inverse correlation between the BI score at baseline and the number of consultation and coordination contacts (see Fig. 2). The lower the BI score, the higher the number of contacts documented.

**Fig. 3:**
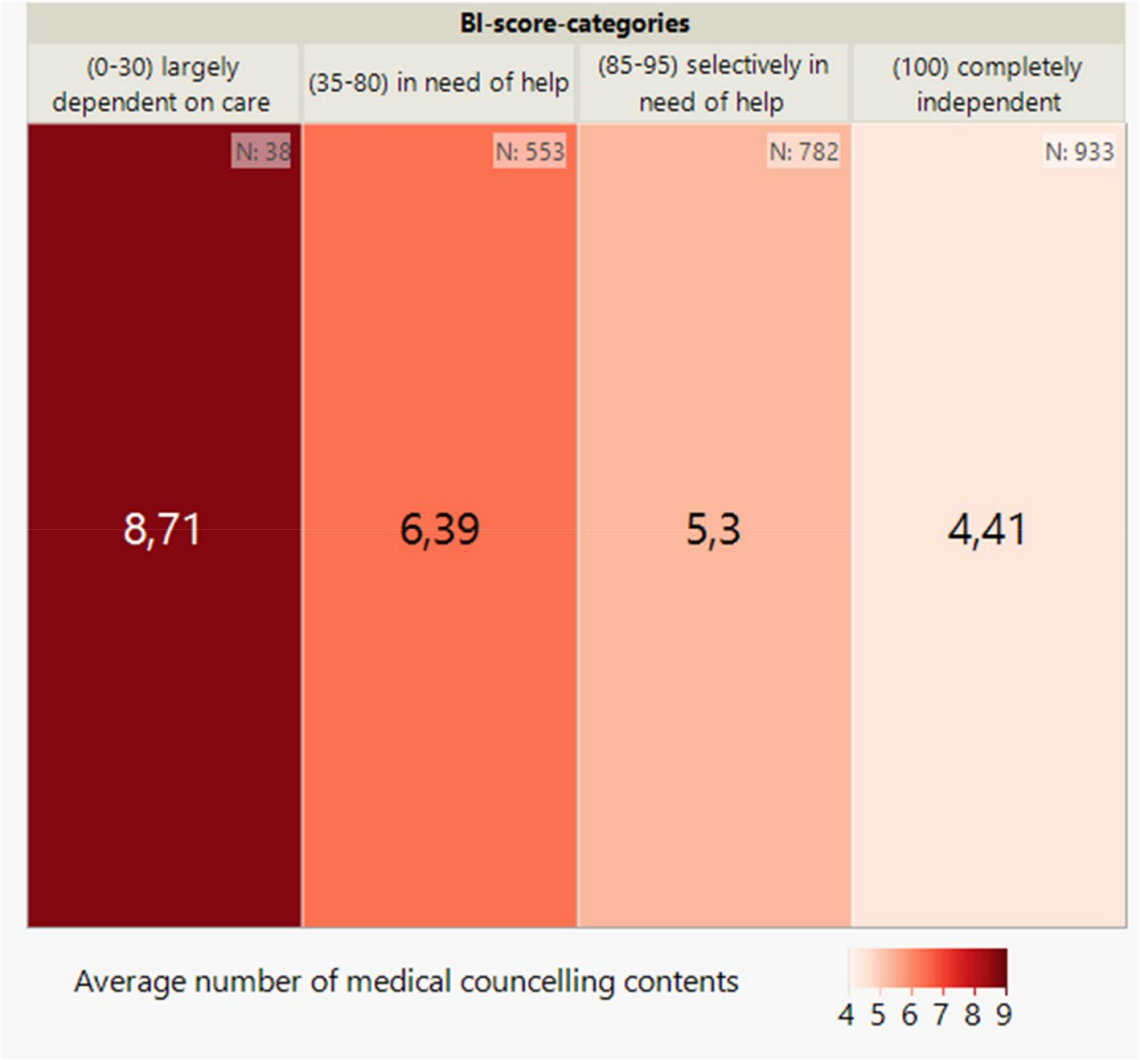
Average number of medical counselling contents per patient by BI score (categorised) at baseline (*n* = 2,306) shown on a heatmap

**Fig. 4:**
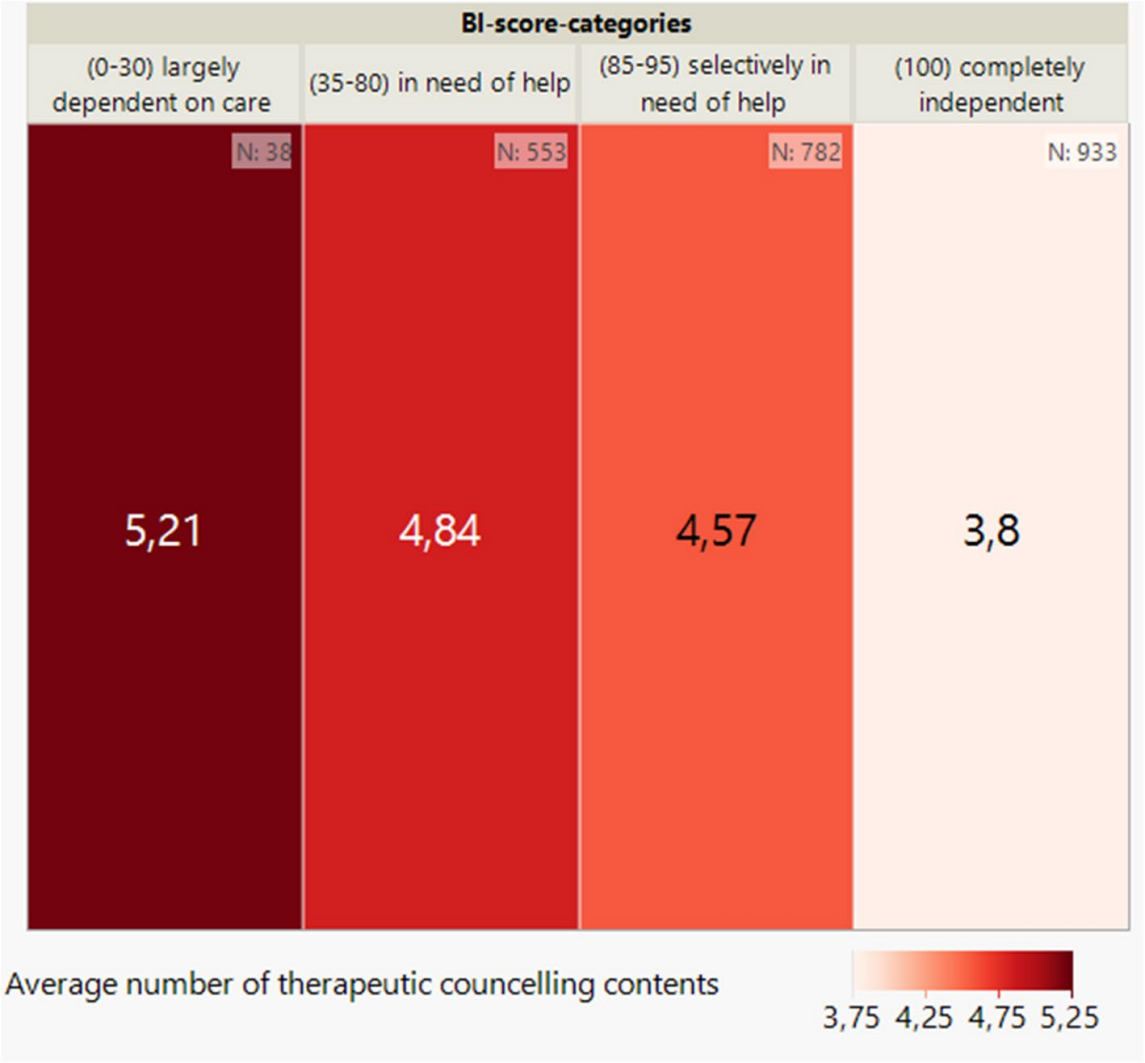
Average number of therapeutic counselling contents per patient by BI score (categorised) at baseline (*n* = 2,306) shown on a heatmap

**Fig. 5:**
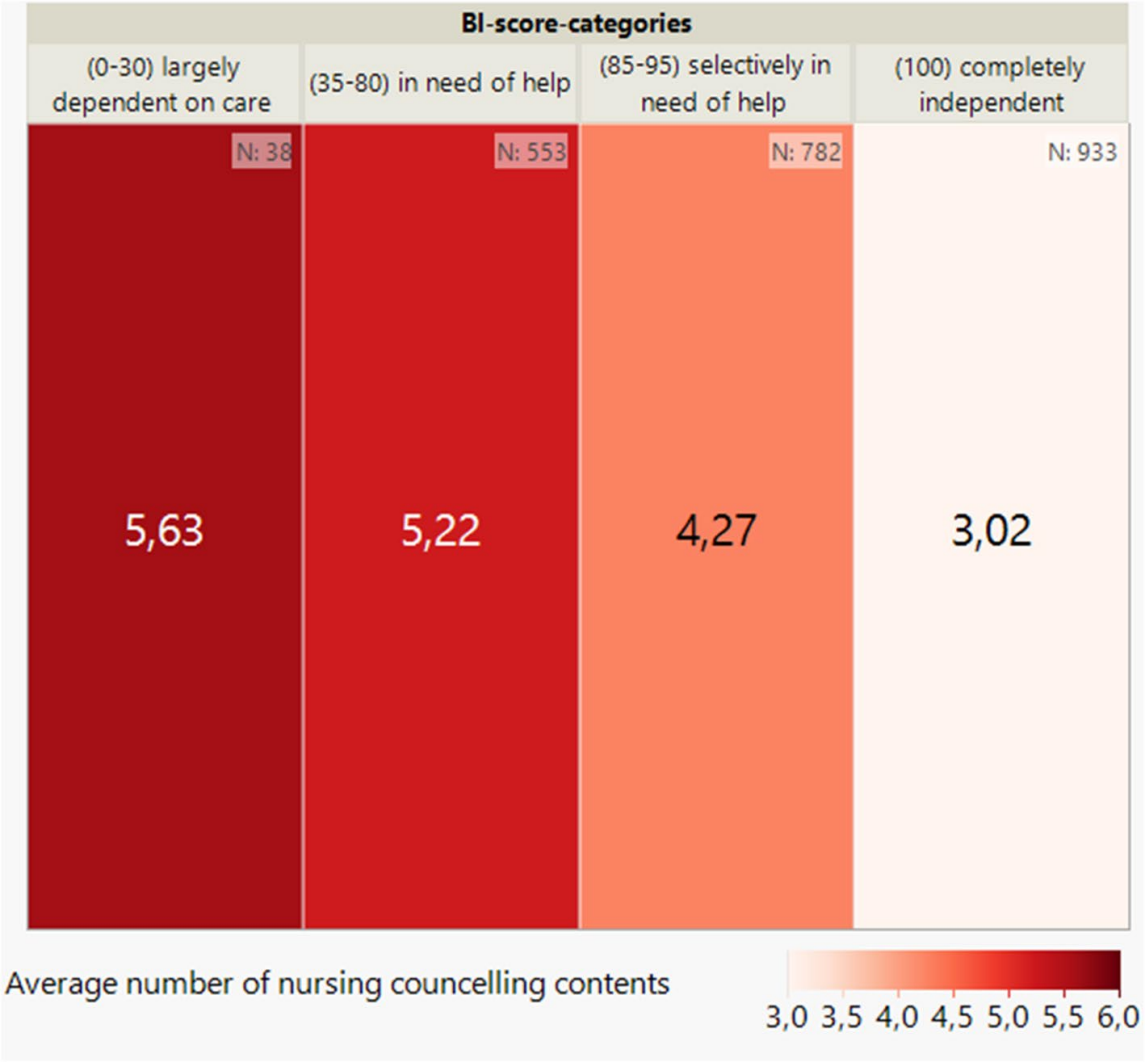
Average number of nursing counselling contents per patient by BI score (categorised) at baseline (*n* = 2,306) shown on a heatmap

**Fig. 6:**
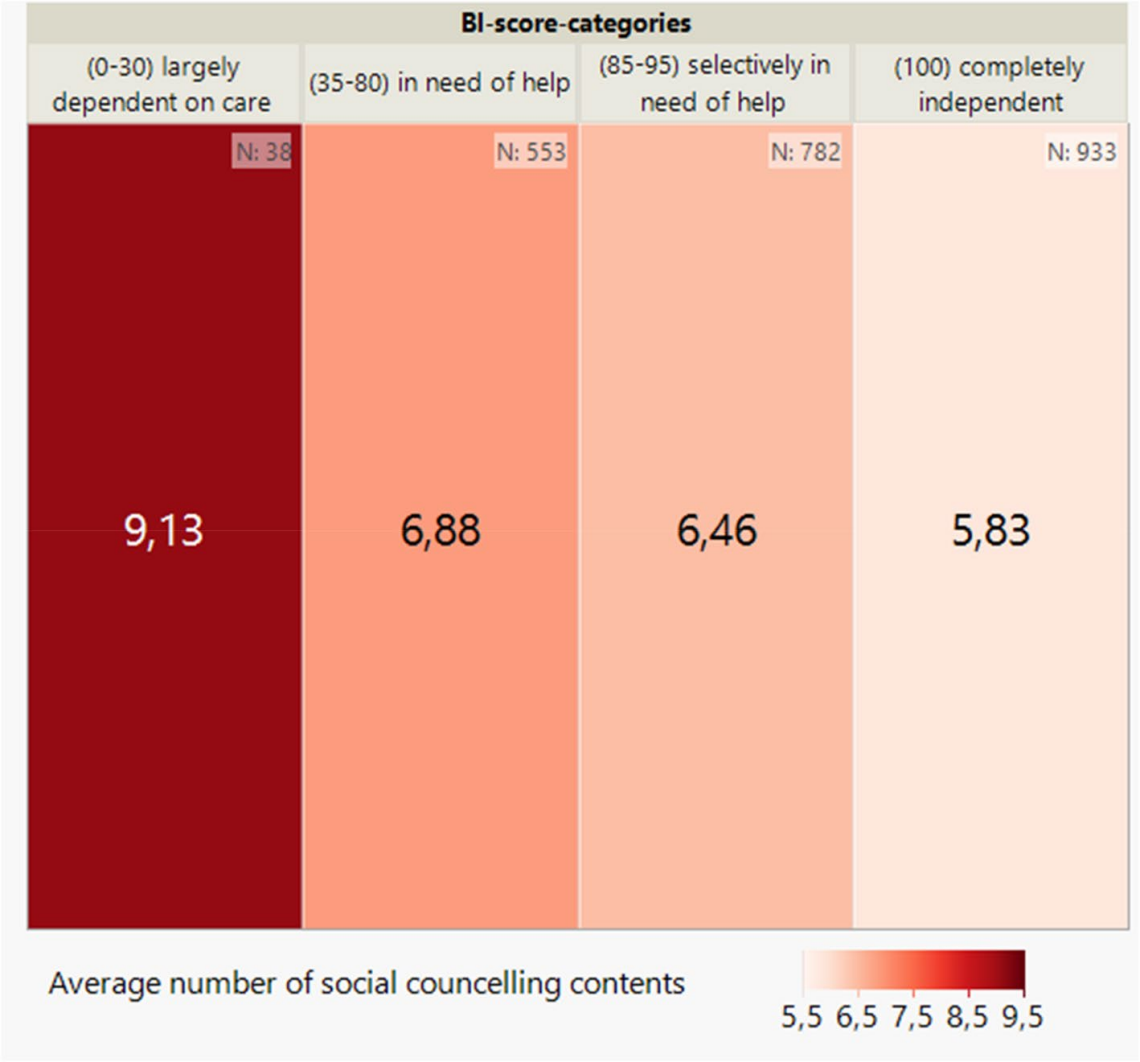
Average number of social counselling contents per patient by BI score (categorised) at baseline (*n* = 2,306) shown on a heatmap

Looking at the distribution of the respective medical, therapeutic, nursing and social counselling content across the BI score categories (see Figs. 3, 4, 5 and 6) per patient, it can be seen, as in Fig. 2, that there is also an inverse relationship here. The lower the BI score or BI score category, the higher the number of counselling contents documented.

### Regression analysis

A multiple linear regression model was used to analyse whether the BI score of geriatric patients influences the intensity of CCM, and which other determinants influence it. The dependent variable was the number of counselling and coordination services provided per patient. The model included 9 potential predictors (independent variables): BI score, age, gender, education (school, profession), living situation (place of residence, home situation), social support and financial situation. The results of this model are presented in Table 4.

**Table 4:** Results of the multiple linear model to analyse the influence of the selected determinants on the intensity of CCM

Variables (reference)	Estimator	[95%-CI]	Std.-Error	t-value	Probability >|t|
ADL-Score					
Barthel-Index-score (0–100)	-0.089007	[-0.11; -0.07]	0.011262	-7.90	< 0.0001*
Place of residence					
Rural (vs. urban)	-1.110256	[-1.48; -0.74]	0.189855	-5.85	< 0.0001*
Home situation					
Living allone (vs. not living allone)	1.0879082	[0.70; 1.48]	0.199437	5.45	< 0.0001*
Social support					
Not exists (vs. exists)	0.1479464	[-0.45; 0.74]	0.304185	0.49	0.6268
School graduation					
Lower (vs. higher)	0.4519155	[-0.01; 0.91]	0.235624	1.92	0.0552
Professional graduation					
Lower (vs. higher)	1.05439	[0.57; 1.54]	0.247375	4.26	< 0.0001*
Financial situation					
Not adaquate (vs. sufficient)	-0.645806	[-1.10; -0.19]	0.232349	-2.78	0.0055*
Age groups					
70–79 (vs. ≥ 80)	0.2693918	[-0.11; 0.65]	0.194006	1.39	0.1651
Gender					
Femal (vs. male)	-0.272102	[-0.67; 0.13]	0.204039	-1.33	0.1825

The multiple linear regression model formulated here achieved moderate variance resolution (goodness of fit) with r^2^ = 0.073 (corrected multiple coefficient of determination r^2^_corrected_ = 0.070) [23]; ^*^p *<* *0.05*

The results of the multiple linear regression show that the respective values of the predictors BI score, place of residence, home situation, professional qualification and financial situation allow statistically significant predictions of the number of counselling and coordination services provided per patient. In detail, this means that the correlation between the increase in BI score and the resulting number of counselling and coordination services is significant, and the increase in BI score by one point reduces the number of counselling and coordination services by 0.089 (*p* = < 0.0001; KI[-0.11; -0.07]). The predictor ‘place of residence’ significantly shows that patients living in rural areas receive fewer counselling and coordination services (-1.1 (*p* = < 0.0001; KI[-1.48; -0.74])) than those living in urban areas. Patients living alone in a private household received more counselling and coordination services (+ 1.1 (*p* = < 0.0001; KI[0.70; 1.48])) than patients not living alone. Patients with a low level of professional qualification were significantly more likely to receive counselling and coordination services (1.0 (*p* = < 0.0001; KI[0.57; 1.54])) than patients with a higher level of professional qualification. It is also significant that patients who were dissatisfied with their financial situation received fewer counselling and coordination services (-0.7 (*p* = 0.0055; KI[-1.10; -0.19])) than patients who were satisfied with their financial situation.

The predictors ‘social support’, ‘education’, ‘age group’ and ‘gender’ had no significant influence in predicting the intensity of CCM in terms of the number of counselling and coordination services.

## Discussion

The central question guiding this investigation is whether it is feasible to predict the necessity for care and case management for geriatric patients who continue to reside in their own homes by ascertaining limitations in activities of daily living using the BI survey instrument. The analysis demonstrated that the BI score documented at baseline in geriatric patients participating in the RubiN study is a suitable indicator for identifying and predicting individual needs for counselling and coordination services of the CCM utilised in the intervention group at an early stage, thereby facilitating effective planning. The Barthel Index is particularly effective because it is simple, reliable and directly related to functional independence. Other assessment tools, such as the Clinical Frailty Scale (CFS) [24] or the Mini-Mental State Examination (MMSE) [25], can provide additional valuable information to quickly and effectively assess the support needs of geriatric patients. Consequently, it is logical to undertake the needs assessment with the BI, complemented by a comprehensive geriatric assessment, in order to ascertain the necessity for counselling and coordination for each individual patient from the outcomes of consultations between GPs, geriatric patients and case managers [26]. For example, measures such as home care, physiotherapy, the provision of medical aids or social support can be specifically addressed by the CCM. Predictable service delivery also allows for more efficient use of resources. This means that patients with high support needs can be referred to care facilities, social services or family care at an early stage, while patients with fewer limitations can be supported with less intensive measures such as fall prevention or home help. This improves the quality of care and reduces unplanned emergencies or hospital admissions. Overall, this supports and enables early, needs-based planning, efficient use of resources and optimal care in the patient’s own home.

The multiple linear regression model identified other factors, in addition to BI, that significantly influenced the intensity of CCM care for geriatric patients. These factors include ‘place of residence’, ‘living situation’ and ‘financial situation’ of the geriatric patients (see Table 4). Patients living in rural areas received fewer consultation and coordination services than those living in urban areas. Studies of differences in regional health care provision have shown that there is evidence of ‘misuse’ in both rural and urban areas. In some cases, there is underuse in rural areas and overuse in urban areas [27]. There may be several reasons for the lower number of contacts with case managers for rural patients in this analysis. It needs to be further investigated whether there are barriers to access or whether, for example, patients living in rural areas develop alternative structures to receive support and advice and therefore make less use of the counselling and coordination services provided by CCM. In this context, it is also important to consider patients’ affiliation to the physician network. Here, it is important to analyse why the number of contacts between case managers and patients differed when the patients’ BI scores in the physician networks were the same.

The fact that a patient lives alone has resulted in a higher frequency of contact from the care and case manager compared to patients who do not live alone. Older people who live alone are often very isolated, lack accessibility in their home environment, such as stairs, and are dependent on outside support and assistance. A possible explanation could be that patients living alone had a greater need for counselling and coordination because they had a higher backlog in this respect than patients not living alone at the start of the RubiN study.

It was also shown that patients who described their financial situation as ‘inadequate’ received fewer contacts from the care and case manager than patients who answered ‘adequate’ to the question about their financial situation. Social status, and therefore living conditions themselves, are relevant to patients’ health, but they also influence patients’ health-related behaviour [28]. Particularly for older and socially disadvantaged people, behavioural interventions in their immediate environment are of great importance, as their spatial radius of action is often limited due to age-related functional limitations, but also due to a lack of financial resources [28]. CCM play a special role in counteracting the negative effects of social inequalities on the health and health behaviour of geriatric patients [29]. The lower use of counselling and coordination services by financially ‘underserved’ patients may indicate a lack of care with regard to relationship-related measures that was not sufficiently identified and could not be compensated by CCM.

### Limitations

The physician network regions were not fully comparable to each other, and the documentation of counselling and coordination services provided by care and case managers was not always standardised.

### Strengths

One of the strengths of this project is the large number of patients available for the analysis. In addition, the study took place in a real care setting, and because there were few exclusion criteria for the patients who participated in the study, it was possible to represent a typical group of geriatric patients in an ambulant setting. The results are therefore transferable to other regions and patients.

## Conclusion

This study demonstrated the usefulness and importance of a low-threshold assessment using the BI questionnaire for patients over 70 years of age in general practice. Based on the BI score, it is possible to identify functional limitations and resources of geriatric patients in a straightforward manner to get first insights in the specific needs of the patients. This information can then be used to estimate the scope and intensity of CCM, allowing planning.

Although CCM in the RubiN project was based on physician networks, which allowed greater flexibility in the use of case managers and therefore better responsiveness to the different needs of geriatric patients, further work is needed to investigate, for example, how regional differences in the frequency of case management counselling and coordination services can be reduced.

## Data Availability

The final trial dataset will be accessible to the study investigators. Data may be also avalaible for project-related research questions in coordination with the project consortium. With a reasonable request, contact the corresponding author SG.

## Abbreviations

AGnES: Community-Based, e-Health-Assisted Systemic Support for Primary Care
RubiN: Acronym for “continuous care in regional networks”
CCM: Care and Case Management
